# Evaluation of efficacy and safety of subcutaneous acetaminophen in geriatrics and palliative care (APAPSUBQ)

**DOI:** 10.1186/s12904-022-00934-3

**Published:** 2022-03-26

**Authors:** Joe El Khoury, Sani Hlais, Mariana Helou, Marie-Claire Mouhawej, Serge Barmo, Patricia Fadel, Aline Tohme

**Affiliations:** 1 Balsam, the Lebanese Center for Palliative Care, Beirut, Lebanon; 2 Family Medicine Department, Saint Joseph University, Beirut, Lebanon; 3 Division of Emergency Medicine, Department of Internal Medicine, Lebanese American University School of Medicine, Beirut, Lebanon; 4 Internal Medicine Department, Palliative Care Team, Hôtel-Dieu de France Hospital, Saint Joseph University, Beirut, Lebanon; 5 Internal Medicine Department, Hôtel-Dieu de France Hospital, Saint Joseph University, Beirut, Lebanon; 6 Clinical instructor, Division of Geriatrics, Saint Joseph University, Beirut, Lebanon

**Keywords:** Paracetamol, Acetaminophen, Subcutaneous injection, Palliative care, Nursing home, Fever, Patient security, Efficacy

## Abstract

**Introduction:**

Subcutaneous infusion (SC) or hypodermoclysis is an old perfusion technique that is often used off-label although it has been shown to be effective. Acetaminophen (paracetamol) subcutaneous injection is performed in some centers despite the lack of conclusive evidence on its effectiveness. This study aims to evaluate the efficacy of subcutaneous infusion of Acetaminophen in the treatment of pain and fever in geriatrics and in palliative care and to determine its safety profile and possible side effects.

**Material and methods:**

This experimental study was conducted between 2018 and 2019 on adult patients in palliative care or in geriatrics in several hospitals and nursing homes in Lebanon. Primary outcomes were change in temperature; change in pain score on the numerical rating scale (NS) or on the Algoplus scale after 60 min from the start of the infusion; and the appearance of local side effects at the infusion site. Changes in the various parameters at 30 min and 180 min were also evaluated as secondary outcomes.

**Results:**

Thirty-one patients were included in the study, with a total of 120 doses of acetaminophen. At 60 min, the mean decrease in pain on the NS was 5.35 points, while the mean decrease in temperature was 0.79 degrees Celsius. At 60 min, 75%, CI = [47.62-92.73] of the patients who received acetaminophen for pain and 66.67%, CI = [38.38-88.17] of those who received it for fever had clinically significant improvement. The overall effect of subcutaneous acetaminophen, defined as any clinically significant effect on pain or fever, was 70.97%, CI = [51.96-85.78]. The overall effect at 30 min and at 180 min was 23.33%, CI = [9.93-42.28] and 87.10%, CI = [70.17-96.37], respectively. The side effects reported 30 min after the injection and observed after at least one of the doses were: local edema in 16 patients (51.61%), induration in one patient (3.23%), local pain in one patient (3.23%) and local heat in one patient (3.23%). At 180 min, only 2 patients (6.45%) still had edema at the infusion site.

**Conclusion:**

Subcutaneous administration of acetaminophen is effective and well tolerated in geriatric and palliative care patients. It is appropriate when no other route is available, especially for home-based care. Comparative studies are needed to allow the expansion of this practice.

## Introduction

Subcutaneous infusion (SC) or hypodermoclysis is an old technique that is often used off-label, although it has been shown to be effective. It is mainly used in palliative care and in geriatrics for its ease of use or when a venous line is no longer available [1, 2]. It is a simple and comfortable technique that allows the delivery of fluids or drugs continuously or discontinuously through the subcutaneous tissue [3–5].

Since many palliative care patients prefer to be cared for and die at home [6–8], hypodermoclysis is a simple and safe technique that allows caregivers to provide care with less constraints and minimal technical support, and ensure that patients remain independent [9–13].

While a good level of scientific evidence for the use of the subcutaneous route is available for a number of molecules from different therapeutic classes, evidence still lacks for some widely used drugs, such as acetaminophen (paracetamol, APAP). Although the efficacy of acetaminophen as an intravenous (IV) analgesic and antipyretic is well known, its use remains controversial through the subcutaneous route. Some teams, especially in France, have nevertheless integrated its use in their daily practice [14, 15]; however many are still reluctant due to the fears of inefficacy or side effects.

Given the lack of established scientific evidence for the use of acetaminophen by this route, our study aims to (1) assess the efficacy of an acetaminophen subcutaneous infusion in the treatment of pain and fever in geriatrics and palliative care patients; (2) determine its safety profile and possible adverse reactions; (3) determine certain characteristics related to this route of administration (early effect, sustained effect over time, etc.).

## Material and methods

### Study design and population

This experimental study was carried out between 2018 and 2019 on geriatrics and palliative care patients who were hospitalized in several hospitals, nursing homes and geriatric centers in Lebanon.

The study was approved by the Institutional Review Board of Hôtel-Dieu de France Hospital (CEHDF 1065) and registered on ClinicalTrials.gov under registration number NCT03635684, first registered on 17/08/2018.

To be included in the study, hospitals and nursing homes staff had to be already routinely using the subcutaneous route for drug or fluid infusions. All nurses involved received adequate training to standardize the preparation of the subcutaneous hypodermoclysis infusion and to report the results.

Adult patients seen by the palliative care consultation team or admitted to the palliative care unit of a university hospital, and geriatric (aged 65 and above) residents of participating nursing homes were included, provided they experienced pain or fever requiring the administration of acetaminophen in the absence of an IV line.

Each patient was informed of the course of the study and signed an informed consent form for their inclusion and use of their data. When the patient was unable to communicate, a legal representative was informed about the study and signed the consent.

A specific subcutaneous route was set up for the exclusive infusion of paracetamol. All other drugs or subcutaneous fluids, if prescribed, were administered via an alternate infusion site to limit drug mixing and subsequent bias.

The subcutaneous route was installed at least 6 hours before administration of the first dose of acetaminophen in order to avoid attributing to acetaminophen some signs or symptoms of discomfort that could be related to the puncturing procedure itself. If a dose had to be given urgently or before the 6-h delay, it was given on a separate injection site and was not taken into consideration in the study. Acetaminophen, available in vials containing 1 g in 100 mL of solution, was infused at a rate of 5 mL/minute (total dose infused over 20 min). To limit bias that could be due to a personal sensitivity (or resistance) to acetaminophen, we estimated that if a patient was receiving 3 doses per day, we would include him/her for a maximum of 1 week, which corresponds to 21 doses. Once this limit was reached, the patients were excluded from the study.

If the patient could receive several analgesics of different classes, the choice between acetaminophen and other analgesics was made according to the usual local protocol in the center where the patient was admitted. Collected data was recorded on daily collection sheets that were dated and signed by the nurse in charge. If an IV route was subsequently installed in a patient, the acetaminophen-specific subcutaneous route could be maintained, and data could continue to be collected following the approval of the treating physician and the consent of the patient.

The onset of local side effects was managed according to the same protocol applied in the department for an IV route.

### Primary endpoints

The main endpoints were a change in temperature or pain scores and the onset of local side effects.

Regarding pain scores, for conscious and cooperative patients, the primary outcome was defined as a change of the pain score on the numerical evaluation scale (NS), 60 min after the start of the infusion. This measure evaluates the decrease in pain scores between t0 (right before acetaminophen administration) and t60 using the NS, where 10/10 is the worst imaginable pain and 0/10 equals no pain. A minimal clinically important difference (MCID) of 2/10 was used following a review of the literature [16, 17].

For patients with verbal communication difficulties, the Algoplus Pain Scale was used to evaluate the decrease in pain scores between t0 (right before paracetamol administration) and t60. The Algoplus scale is a behavioral rating scale for acute pain in patients with verbal communication difficulties, where a score of at least 2 over 5 can diagnose pain with a sensitivity of 87% and a specificity of 80%. The score assesses facial expressions, appearance, complaints, body position and atypical behaviors of patients with verbal communication difficulties. Each item marked “yes” is given one point. For the purpose of this study, effectiveness was defined as a decrease in score from ≥2/5 to less than 2/5 [18, 19].

For patients presenting with fever, a decrease in temperature 60 min after the start of the infusion with an MCID of 0.5° Celsius was defined as the primary outcome.

A composite result for the overall effect on pain at 60 min, combining the results collected using the NS and the Algoplus scale, as well as a composite result for the overall effect on pain and fever, were computed.

Local adverse effects at the infusion site were reported, including: edema, induration, erythema, tenderness, heat, abscess, necrosis. Any other local side effects were noted by the nurses. Side effects were assessed before the first injection as well as 30 min, 1 h and 3 h after each injection. They were also evaluated 24 h after removal of each of the sites of subcutaneous administration of acetaminophen.

Changes in pain scores and temperature at t30 and t180 were evaluated as secondary outcomes and a composite result for an overall effect on pain at 30 min, as well as a result for the overall effect at 180 min, were created.

Several factors likely to influence the occurrence of local side effects or the effectiveness of the drug were gathered: age, sex, weight, use of other analgesic drugs, total number of paracetamol injections, injection site, pain etiology (cancer or non-cancer pain), cause of fever (localized infection, sepsis or paraneoplastic), acetaminophen manufacturer, and inclusion center.

### Statistical analysis

Statistical analysis was carried out using STATA version 10 software, the quantitative variables being represented by their means and standard deviations, the qualitative variables by their numbers and percentages.

Sample size was calculated based on an expected efficacy on pain of 36% [20], with a margin of error of 5% and an alpha error of 5%; it was estimated at 255 doses of acetaminophen.

To determine the effectiveness of the treatment, the following calculations were performed:

For patients in the NS group, the mean change per patient at 30 min, 60 min, and 180 min was calculated to determine whether the infusion was overall effective for that patient. For example, if the mean change at 60 min on the NS, all doses combined, for a patient was greater than or equal to 2, paracetamol was deemed effective for that patient after a period of 60 min. If the mean change at 60 min was less than 2/10, the infusion was considered ineffective in this patient.

For patients in the Temperature group, the mean change in fever per patient at 30 min, 60 min, and 180 min was calculated to determine whether the infusion was overall effective for that patient. For example, if the mean change at 60 min, all doses combined, for a patient was greater than or equal to 0.5 degrees Celsius, paracetamol was judged effective for that patient after a period of 60 min. If the mean change at 60 min was less than 0.5 degrees Celsius, the infusion was considered ineffective in this patient.

For patients assessed using the Algoplus scale, at least half of the doses received by the patient had to be effective (thus causing a decrease in the Algoplus score to less than 2/5) in order to consider paracetamol effective for this patient, at 30 min, 60 min and 180 min.

## Results

Thirty-one patients were included in the study, with a total of 120 doses of acetaminophen administered.

The main characteristics of the patients are summarized in Table 1.

**Table 1:** Sample characteristics

Characteristics		Number (%)	Quantitative variables
Age			Mean: 83.3
			Standard deviation: 9.67
			Median: 83.5
			Minimum: 54
			Maximum: 98
Gender	Male	5 (16.13)	
	Female	26 (83.87)	
Inclusion center	Hôtel-Dieu de France	2 (6.45)	
	Maison Notre Dame	17 (54.84)	
	Age Optimum Geriatric Center	8 (25.81)	
	Saint Florian (Bhannes Hospital)	1 (3.23)	
	Notre Dame Hospital	3 (9.68)	
Weight	< 50 Kg	9 (29.03)	
	> 50 Kg	22 (70.97)	
Patients with other prescribed analgesics		8 (25.81)	
Indication	Pain		
	*Non-cancer*	11 (35.48)	
	*Cancer*	5 (16.13)	
	Fever		
	*Local infection*	11 (35.48)	
	*Sepsis*	3 (9.68)	
	*Paraneoplastic*	1 (3.23)	
Total number of acetaminophen doses given		120	
Acetaminophen brand	Perfalgan	120 (100)	
Doses per patient			Mean: 3.9
			Standard deviation: 5.21
			Median: 2
			Minimum: 1
			Maximum: 21
Injection site	Thigh	120 (100)	
Pain scale	Numerical scale	8 (50)	
	Algoplus	8 (50)	
Cause for stopping data collection	Symptoms resolution	12 (38.71)	
	Home discharge	4 (12.90)	
	Death	6 (19.35)	
	IV line installation	1 (3.23)	
	Line torn away by agitated patient	2 (6.45)	
	Maximum number of doses reached	1 (3.23)	
	Spontaneous disinsertion of the SC line	2 (6.45)	
	Analgesic treatment change	1 (3.23)	
	Missing data	2 (6.45)	

For all patients included and all doses combined, at 60 min, the mean decrease in pain on the NS was 5.35 points, and the average decrease in temperature was 0.79 degrees Celsius. At 180 min, the mean decrease was 6.23 points and 1.35 degrees Celsius, respectively.

On the Algoplus scale, for all patients and all doses combined, 2.86% of doses were effective at 30 min (reduction in the score to less than 2/5), 80% at 60 min, and 88% at 180 min. (Table 2).

**Table 2:** Improvement following the infusion of subcutaneous paracetamol, all doses combined: descriptive data

Scale	Numerical Scale (*N* = 51-52)	Temperature (*N* = 30-32)	Algoplus (*N* = 35-36)
	Average decrease (/10)	Average decrease (Degrees Celsius)	Percentage of effective doses (%)
30 min	4.46	0.26	2.86
60 min	5.35	0.79	80
180 min	6.23	1.35	88

At 60 min, 12 (75%, CI = [47.62-92.73]) of 16 patients who received acetaminophen for pain had clinically significant improvement in their symptom, 10 (66.67%, CI = [38.38-88.17]) of 15 patients who received acetaminophen for fever had clinically significant improvement of their fever. The overall effect of subcutaneous acetaminophen, defined as any clinically significant effect on pain or fever, was 70.97%, CI = [51.96-85.78].

Among the patients who received acetaminophen for pain relief, only 3 (20%, CI = [4.33-48.09]) had a clinically significant improvement after 30 min and 14 (87.5%, CI = [61.65-98.45]) had a prolonged effect after 180 min.

Among the 15 patients who received acetaminophen for fever, 4 (26.67%, CI = [7.79-55.6]) showed a clinically significant improvement after 30 min and 13 (86.67%, CI = [59.54-98.63]) had a prolonged effect after 180 min.

**Fig. 1:**
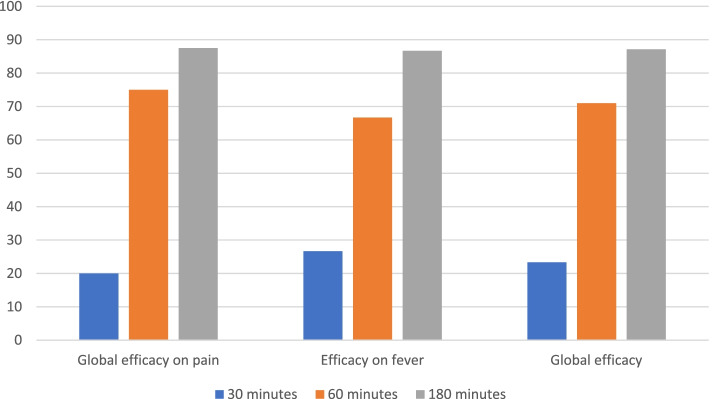
Evolution of the efficacy of acetaminophen subcutaneous infusion over time

The overall effect of subcutaneous paracetamol at 30 min and 180 min was 23.33% (CI = [9.93-42.28]) and 87.10% (CI = [70.17-96.37]), respectively. (Table 3 and Fig. 1).

**Table 3:** Primary and secondary results. Effectiveness of acetaminophen subcutaneous infusion according to the indication and different scales. n: number of patients. NS: Numerical Scale. CI: Confidence interval

	Efficacy on pain			Efficacy on fever	Global efficacy (*n* = 31)
	NS (***n*** = 8)	Algoplus (***n*** = 7 or 8)	Global (***n*** = 16)	(***n*** = 15)	Pain and fever combined
*Primary Outcomes- % [CI]*					
At 60 min	62.5	87.5	**75 [47.62-92.73]**	66.67 [38.38-88.17]	**70.97 [51.96-85.78]**
*Secondary outcomes- Number (%) [CI]*					
At 30 min	25	14.29	**20 [4.33-48.09]**	26.67 [7.79-55.6]	**23.33 [9.93-42.28]**
At 180 min	75	100	**87.5 [61.65-98.45]**	86.67 [59.54-98.63]	**87.10 [70.17-96.37]**

Adverse reactions reported 30 min after injection following at least one of the doses were local edema in 16 patients (51.61%), induration in one patient (3.23%), pain in one patient (3.23%) and local heat in one patient (3.23%).

At 180 min, only 2 patients (6.45%) still had edema at the infusion site and no other side effects were reported at this time. In all cases, edema was described as minimal by the nurses.

**Fig. 2:**
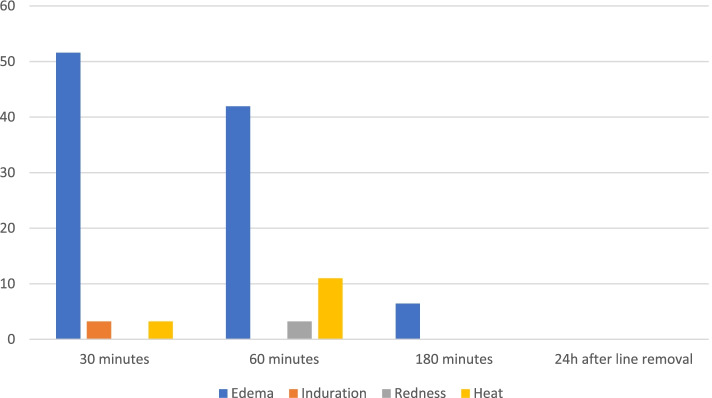
Evolution of the prevalence of side effects over time following subcutaneous Acetaminophen infusion

No adverse events were reported the day after SC line removal. (Table 4 and Fig. 2).

**Table 4:** Prevalence of adverse reactions following at least one dose at each time interval. N: number of patients

Adverse reaction following at least one dose	30 min N (%)	60 min	180 min	24 h after line removal
Edema	16 (51.61)	13 (41.94)	2 (6.45)	0 (0)
Induration	1 (3.23)	0 (0)	0 (0)	0 (0)
Redness	0 (0)	1 (3.23)	0 (0)	0 (0)
Pain	1 (3.23)	0 (0)	0 (0)	0 (0)
Heat	1 (3.23)	2 (6.45)	0 (0)	0 (0)
Abscess	0 (0)	0 (0)	0 (0)	0 (0)
Necrosis	0 (0)	0 (0)	0 (0)	0 (0)

The main reason for stopping the infusion was resolution of symptoms (38.71%), followed by death (19.35%) due to baseline illness. In one patient, the maximum number of doses allowed by the study protocol was reached (21 doses) and data collection was therefore stopped, although the infusion was continued at the request of the patient who was satisfied with the effectiveness of the treatment.

## Discussion

This study is the first to our knowledge to have evaluated and demonstrated the efficacy of acetaminophen subcutaneously. Our results showed overall efficacy in nearly 71% of cases as well as, all doses and all patients combined, an average pain reduction of 4 to 6 points on the numerical scale (at 60 min and 180 min), and an average fever decrease of 1.3 degrees Celsius sustained at 180 min.

The efficacy of acetaminophen as an analgesic and IV antipyretic has long been demonstrated [21–27], and unlike many other pain relievers, it has a favorable safety profile even in infants [28]. Our results on pain are in agreement with the available data on the efficacy of IV, oral and rectal acetaminophen. Indeed, a study conducted by Nichols et al. published in 2016 demonstrated an average reduction in pain of nearly 3 to 4 points on the numerical scale following the administration of IV or oral (PO) paracetamol, with an earlier effectiveness with the IV formulation (significant difference at 30 min compared to oral administration) [29]. Similar results were found in several studies on IV acetaminophen, grouped together in a review of the literature published by Duggan et al. [26], while a 2008 meta-analysis confirmed that rectal and IV acetaminophen have similar efficacy on pain and fever in children [30].

Our results show a higher efficacy of subcutaneous acetaminophen compared to a 2016 Cochrane review (71% versus 36%, respectively) [20]. There are major differences between the two: Our study analyzed the effect of acetaminophen on pain and fever, was patient-centered, and included patients with diverse pain and fever etiologies, while the Cochrane review only focused on pain, was dose-centered, and included exclusively postoperative patients. Additionally, NS pain outcome definitions are different (2-point decrease in our study versus 50% decrease in the review), and it is unknown if average baseline pain levels were similar in the two studies. These are all possible explanations to the difference between our results and the reviews’, as literature shows that acetaminophen efficacy is influenced by baseline pain intensity and the origin of the pain [31].

Our results also show a higher average decrease on the NS than other published studies. Although we do not have a definitive explanation to this finding, we understand that the pain experience and rating can vary widely across cultures. Even within the United States, it was noted that African Americans and Hispanics tend to report higher pain scores when compared to Whites and Asians [32]. As our sample only included Lebanese participants, we decided to present the average changes as descriptive results only, and consider any decrease of at least 2 points on the NS as a positive result without analyzing the range of decrease.

The effect of IV acetaminophen on fever was evaluated in a double-blind non-inferiority study comparing the efficacy of a single dose of intravenous acetaminophen at 15 mg/kg (*n* = 33) with that of propacetamol at 30 mg/kg (*n* = 32) in the treatment of pediatric patients with acute fever due to infection [33]. Propacetamol is an acetaminophen bioprecursor, used for the same indications. Intravenous acetaminophen was found to be non-inferior to intravenous propacetamol in reducing mean peak body temperature (reduction of 1.92 vs. 2.05 °C; difference between groups 0.13 degrees Celsius [95% CI - 0.25, 0.54]) [33]. Our results using the subcutaneous route seem inferior, while maintaining a clinically significant efficacy.

Usually, the maximum efficacy of drugs with first-order pharmacokinetics, when injected subcutaneously, is reached 30 min after the time of injection [34]. Many analgesics, including morphine and other opioids, follow this pharmacokinetics [34, 35]. In our study, the efficacy of acetaminophen was delayed and started 60 min after the injection. This could be explained by the large volume of the product (100 mL) which accumulates subcutaneously and is gradually absorbed, as well as by the prolonged infusion rate (20 min). In comparison, the maximum volume for subcutaneous injections is generally set at only 2 mL to allow good absorption and efficacy [36].

The action of subcutaneous acetaminophen is sustained in our study for at least 3 h after injection. These results agree with the data on oral and IV  acetaminophen in the literature [26].

Given the small number of patients included, this study does not have the necessary power to assess patients’ subclasses and correlate efficacy and/or side effects with different demographics or symptoms etiologies leading to the prescription of acetaminophen.

We note, however, that the only patient who had no improvement in her temperature had paraneoplastic fever. In addition, it appears that the presence of edema of the lower extremities in another patient limited the effectiveness of the infusion, as the results seemed to be better following resolution of the edema.

A good level of scientific evidence for the use of the subcutaneous route is available for a number of molecules from different therapeutic classes, which allows their frequent use in geriatrics and palliative care [1, 2, 34, 35, 37–41]. Our results on acetaminophen seem to converge towards this same conclusion.

Regarding tolerability, this study demonstrated the safety of the subcutaneous injection of acetaminophen. Throughout the study, no patient experienced a serious side effect, whether ulceration, necrosis, or abscess. Edema was the main adverse effect observed, which is in agreement with the perception of prescribers of this practice and with the results found by a team of French researchers led by Dr. Leheup during a prospective observational study published in July 2018 [14, 15]. One of the advantages of our study over the study conducted by Dr. Leheup [14] is the exclusive use of a specified infusion site for acetaminophen, thus limiting the bias that may be related to the mixing of different solutions in the tubing or the infusion site. We note a rapid resolution of the edema, attributable to deposition and then absorption of the accumulated product in the subcutaneous area.

Local edema as a side effect of subcutaneous infusions is often found in the literature, and no serious consequences related to this phenomenon have been reported [2, 14, 41].

These results are promising, given the multiple benefits of the parenteral formulation of acetaminophen compared to the oral one. It has a faster onset of action and a higher maximum serum concentration [26]. For an equivalent dosage, acetaminophen administered intravenously also has a higher and more stable plasma and cerebrospinal fluid (CSF) concentration than the PO and rectal formulations [42], and this could be extrapolated to subcutaneous administration. There are no published studies comparing the pharmacokinetics of IV versus subcutaneous acetaminophen, but a randomized trial was ongoing at the time of writing of this article [43]. Another advantage of subcutaneous acetaminophen is that its absorption is not affected by opioids. Oral acetaminophen absorption is reduced due to delayed gastric emptying caused by opioids, as shown in a study comparing serum acetaminophen concentrations in patients treated with acetaminophen + morphine or acetaminophen + ketorolac after surgery [44]. Gastrointestinal absorption of oral pain relievers may also be reduced by fasting, anesthesia, and patient stress after surgery [44, 45]. In view of these advantages, subcutaneous acetaminophen could be used in patients who cannot tolerate oral medications (eg with swallowing disorders, vomiting, gastrointestinal motility disorders), patients who do not respond to oral pain relievers and those with moderate to severe uncontrolled or acute pain. Moreover, given its ease of administration, it could constitute a simple and safe alternative which would allow caregivers in an outpatient environment (for example at home) to provide care with less constraints, minimal technical support, and ensure that patients retain a better autonomy [9–13]. Paracetamol infusion is currently expensive in the USA, which limits its use in this context, but it is very cheap and available in Europe [26].

Nowadays, when patients fail to tolerate the oral route and in the absence of an IV route, many clinicians would adopt the rectal administration of acetaminophen suppositories before thinking of the subcutaneous route. Rectal route can be limited by cultural acceptance (especially in Lebanon) as well as medical conditions like hemorrhoids and anorectal diseases like anal ulcers and abscesses. Our results are encouraging as they may help spare patients the disagreements of a rectal administration, in terms of physical discomfort (especially in patients who experience pain with position changing) and psychological distress.

This study has limitations. First, the small number of included patients and doses which could have led to an overestimation of the effectiveness or the incidence of side effects of the treatment, as well as the inability to conduct subgroup analyses. We faced two major difficulties, one being the low cooperation and engagement from the local prescribing physicians in some centers, who despite our repetitive visits and reminders, did not consistently propose inclusion into our study to their patients; the second being the presence of an IV line or a port-a-cath in most hospitalized patients, which was an exclusion criterion in our study following the requirement of the ethics committee. In Lebanon, the hospital system is heavily dependent on private insurers, who refuse to keep paying for a hospital stay if there is no need for IV treatments – Thus the systematic insertion of an IV line to virtually all patients as soon as they are admitted. This constituted a major difficulty in finding patients for our study. However, and although we could not hit our target of 255 doses, our results are encouraging and could help predict the sample size of a bigger study in the future.

Although no serious side effects were observed, our study may not have the power to detect rare events and it is therefore possible that they went unnoticed, knowing that our results are similar to those of bigger, multicentric studies [14]. Finally, this study does not include a comparison group and only compares the different parameters according to a “before / after” design. Since the administration of acetaminophen subcutaneously is in practice reserved for patients who either cannot benefit from an intravenous route and in whom the subcutaneous route may be the only choice, or for whom a subcutaneous route is more comfortable and in accordance with their goals of care, such a design was chosen for this pioneering study in order to first prove that the concept seems effective and could be developed. A study comparing subcutaneous acetaminophen with intravenous paracetamol would therefore be of great interest.

## Conclusion

Our study shows that the subcutaneous administration of acetaminophen is effective and well tolerated in geriatric and palliative care patients. It would therefore appear to be appropriate when no other route is available, reliable, or acceptable to the patient, when patient’s comfort is among their goals of care, or when the patient is receiving care at home. However, comparative experimental studies are necessary to allow the expansion of this practice.

## Data Availability

The datasets generated and/or analysed during the current study are available in the Mendeley repository, with the DOI: 10.17632/dmdymrhnfd.1
